# Static and dynamic aspects of cerebro-cerebellar functional connectivity are associated with self-reported measures of impulsivity: A resting-state fMRI study

**DOI:** 10.1162/netn_a_00149

**Published:** 2020-09-01

**Authors:** Majd Abdallah, Nicolas Farrugia, Valentine Chirokoff, Sandra Chanraud

**Affiliations:** Aquitaine Institute of Cognitive and Integrative Neuroscience, UMR CNRS 5287, University of Bordeaux, France; Electronics Department Lab STICC, IMT Atlantique, UMR CNRS 6285, Brest, France; Aquitaine Institute of Cognitive and Integrative Neuroscience, UMR CNRS 5287, University of Bordeaux, France; Aquitaine Institute of Cognitive and Integrative Neuroscience, UMR CNRS 5287, University of Bordeaux, France; Laboratory of Neuroimaging and Daily Life, EPHE, PSL Research University, Bordeaux, France

**Keywords:** Cerebellum, Cerebro-cerebellar system, Impulsivity, Resting-state fMRI, Static functional connectivity, Dynamic functional connectivity

## Abstract

Human and animal brain studies bring converging evidence of a possible role for the cerebellum and the cerebro-cerebellar system in impulsivity. However, the precise nature of the relation between cerebro-cerebellar coupling and impulsivity is far from understood. Characterizing functional connectivity (FC) patterns between large-scale brain networks that mediate different forms of impulsivity, and the cerebellum may improve our understanding of this relation. Here, we analyzed static and dynamic features of cerebro-cerebellar FC using a highly sampled resting-state functional magnetic resonance imaging (rs-fMRI) dataset and tested their association with two widely used self-reports of impulsivity: the UPPS-P impulsive behavior scale and the behavioral inhibition/approach systems (BIS/BAS) in a large group of healthy subjects (*N* = 134, ≈ 1 hr of rs-fMRI/subject). We employed robust data-driven techniques to identify cerebral and cerebellar resting-state networks and extract descriptive summary measures of static and dynamic cerebro-cerebellar FC. We observed evidence linking BIS, BAS, sensation seeking, and lack of premeditation to the total strength and temporal variability of FC within networks connecting regions of the prefrontal cortex, precuneus, posterior cingulate cortex, basal ganglia, and thalamus with the cerebellum. Overall, our findings improve the existing knowledge of the neural correlates of impulsivity and the behavioral correlates of the cerebro-cerebellar system.

## Author Summary

Accumulating evidence from preclinical and neuroimaging studies proposes that the cerebellum regulates impulsive behavior through its interactions with brain regions that subserve control and reward processes. To further explore this proposal, we analyzed static and dynamic aspects of resting-state functional connectivity between the cerebellum and distinct large-scale brain networks using robust methods and evaluated them against a set of self-reported measures of impulsivity. We found compelling new evidence linking multiple forms of impulsivity to strength and temporal variability of FC between large-scale cerebral networks, involved in top-down control and reward, and the cerebellum. Our findings highlight the utility of combining static and dynamic FC approaches in furthering current understanding of cerebro-cerebellar coupling and the neurobiology of complex behaviors.

## INTRODUCTION

Impulsivity is a multidimensional personality construct present to varying degrees in healthy individuals as well as diverse neuropsychiatric populations (Bakhshani, 2014). Psychologists define impulsivity as the tendency to initiate actions dominated by spontaneity and urgency with little to no consideration of consequences (Bakhshani, 2014). Self-report questionnaires are often used to measure two broad constructs highly related to impulsivity: inhibitory control and reward sensitivity (Jauregi, Kessler, & Hassel, 2018). Interindividual differences in these processes are believed to arise from genetic and neural origins that are not fully understood (Khadka et al., 2014). However, extensive evidence from neuroimaging studies suggests that monoaminergic cortico-striatal systems underlie the different constructs of impulsivity (Dalley, Everitt, & Robbins, 2011; Fineberg et al., 2014; Mitchell & Potenza, 2014). Interestingly, a hypothesis for the involvement of the cerebellum and the cerebro-cerebellar system in impulsivity has recently been advanced by Miquel, Nicola, Gil-Miravet, Guarque-Chabrera, and Sanchez-Hernandez (2019) based on numerous findings from human and animal brain studies. These findings propose that the cerebellum regulates different forms of impulsivity by interacting with and modulating regions of the prefrontal cortex and the basal ganglia (Miquel et al., 2019).

Originally thought of as a sensorimotor structure, the cerebellum is now known to be functionally diverse and involved in higher cognitive processes (Buckner, 2013; Strick, Dum, & Fiez, 2009). Studies have confirmed that the majority of the cerebellum maps onto association regions in a manner that mirrors the cerebral asymmetries for cognition, language, and attention (Buckner, 2013; Habas et al., 2009). In this context, consistent findings from many studies indicate that the cerebellum may be involved in higher cognitive processes related to impulsivity (Miquel et al., 2019). Neuroimaging studies have identified structural and functional connections between the cerebellum and brain regions that subserve control and reward brain processes, such as the prefrontal cortex, anterior cingulate cortex, insula, ventral tegmental area, thalamus, and basal ganglia (Caligiore et al., 2017; Carta, Chen, Schott, Dorizan, & Khodakhah, 2019; Moreno-Rius & Miquel, 2017). Moreover, patients with posterior cerebellar damage have been observed to exhibit difficulties in controlling behavior and emotions, and show signs of impulsiveness and behavioral disinhibition (Schmahmann & Sherman, 1997). In addition, certain brain disorders that exhibit alterations in the cerebro-cerebellar circuitry, such as alcohol use disorder, also feature impulsivity as a key component of the disease pattern (Jung et al., 2014; Miquel et al., 2019). Although supported by accumulating evidence, the link between cerebro-cerebellar coupling and impulsivity has not been thoroughly investigated within the framework of functional connectivity (FC). Characterizing the patterns of FC between cerebral networks that mediate cognitive processes such as inhibitory control and reward processing, and the cerebellum, may improve our understanding of the hypothesized cerebellar role in impulsivity.

Resting-state functional magnetic resonance imaging (rs-fMRI) has received attention for the comprehensive evaluation of interregional FC in the absence of tasks (Sporns, 2018; Van den Heuvel & Pol, 2010). It has proven to be suitable for the exploration of the neural correlates of certain behaviors, cognitive abilities, and personality traits in both healthy and clinical populations. Resting-state fMRI studies have uncovered important principles of cerebellar functioning pertaining to the spatiotemporal organization of networks in the cerebellum (Buckner, Krienen, Castellanos, Diaz, & Yeo, 2011; Guell, Gabrieli, & Schmahmann, 2018). Importantly, studies have shown that the cerebellum contains individual-specific representations of most well-known cortical networks including an overrepresented executive control network (Marek et al., 2018). Until recently, most of the rs-fMRI literature have assessed interregional coupling under the assumption of stationary FC, but this “static” approach is believed to miss out on valuable information embedded in the dynamic nature of the brain (Calhoun, Miller, Pearlson, & Adalı, 2014; Lurie et al., 2020). Accordingly, recent studies have begun exploring the temporal dynamics of brain activity and connectivity, and have pointed to the presence of ongoing temporal reconfiguration of FC strength that supports cognition and exhibits alterations in several brain disorders (Allen et al., 2014; Lurie et al., 2020). Measures of dynamic FC complement and, in some cases, outperform measures of static FC in explaining certain behavioral factors (Liégeois et al., 2019). However, joint information from both captures more variance in behavior than either alone (Liégeois et al., 2019; Ramos-Nuñez et al., 2017). Major advances have been made in understanding how cerebral networks dynamically interact and impact behavior, but little is known about the dynamics of cerebro-cerebellar networks and their behavioral correlates. That being said, we believe that exploiting static as well as dynamic aspects of cerebro-cerebellar FC can provide further insight into the functional repertoire of the cerebro-cerebellar system.

In this study, we aimed to characterize cross-sectional differences in static and dynamic cerebro-cerebellar FC, and test for potential associations with self-reported impulsivity. The uniform cytoarchitecture of the cerebellum suggests that cerebellar modules differ in the projection of their afferent and efferent connections to sensorimotor and higher cognitive networks while serving a unitary domain-general function (Marek et al., 2018; Voogd & Glickstein, 1998). Therefore, we sought to estimate the total strength and temporal variability of FC between cerebral networks of interest and the cerebellum. These measures reflected different aspects of the total cerebellar influence within distinct large-scale brain networks. We hypothesized that FC between cerebral networks, which are involved in control and reward brain processes, and the cerebellum could be associated with impulsivity. To test this hypothesis, we employed an open dataset comprising highly sampled resting-state fMRI data (four runs, ≈ 15 min/run) and self-reports assessing different elements of impulsivity, from a large group of healthy participants (*N* = 134, 62 females). We decomposed the rs-fMRI data into separate cerebral and cerebellar resting-state networks (RSNs), using data-driven techniques, to account for the functional heterogeneity present in both structures. Then, we estimated static FC among the identified RSNs and used hidden Markov models (HMMs) to model whole-brain dynamics and estimate subject-specific dynamic FC matrices (brain states; Vidaurre, Smith, & Woolrich, 2017). Finally, we calculated summary measures of the total strength and temporal variability of FC between distinct cerebral RSNs of interest and the cerebellum and evaluated them against self-reported measures of impulsivity using multivariate general linear models.

## MATERIALS AND METHODS

### Participants

A total of 134 healthy participants (62 females, ages 20–40 years) from the Neuroanatomy and Connectivity protocol (N&C), which is part of the Max Planck Institute Leipzig Mind-Brain-Body (MPILMBB) dataset, were included in this study. All included participants were healthy with no past or present signs of any neuropsychiatric condition, fulfilled the MRI safety requirements, and provided written informed consent prior to their participation (Mendes et al., 2019). Originally, the dataset included fully preprocessed MRI data and a battery of behavioral assessments from 188 participants. However, because of a gap in the age distribution, we excluded 26 subjects that were older than 55, and 28 subjects for missing data from source. Details on the inclusion and exclusion criteria can be found in Mendes et al. (2019).

### Self-Reports of Impulsivity

Two widely used self-assessments of impulsivity were included in this study: the UPPS-P impulsive behavior scale and the behavioral inhibition/approach systems scales, widely known as BIS/BAS. The normality of the different variables was tested using the Shapiro-Wilk test, and departures from normality were counteracted using rank-based inverse Gaussian transform. The two self-reports are described in detail in the following paragraphs.

The UPPS-P impulsive behavior scale is a self-report questionnaire designed to measure impulsive behavior across the five-factor model of personality: negative urgency, positive urgency, sensation seeking, lack of premeditation, and lack of perseverance (Whiteside & Lynam, 2001). Higher scores on the negative and positive urgency subscales indicate a higher tendency to act rashly under the effect of negative and positive emotions, respectively. A higher score on the sensation-seeking subscale indicates a higher tendency to seek novel experiences. Furthermore, a higher score on the lack of premeditation subscale indicates a higher tendency to act rashly without planning or thinking. Finally, a higher score on the lack of perseverance subscale indicates an increased inability to remain focused and engaged in a possibly arduous and boring task (Whiteside & Lynam, 2001).

The BIS/BAS scale is also a self-report questionnaire that measures two general motivational systems argued by theorists to underlie behavior: a behavioral inhibition system (BIS) that regulates sensitivity to punishment and negative cues, and a behavioral approach system (BAS) that regulates sensitivity to desirable cues and nonpunishment (i.e., rewards). A higher score on the BIS scale indicates an increased sensitivity to negative outcomes of anticipated actions and hence a higher tendency to avoid them, whereas a higher score on the BAS scale indicates an increased sensitivity to rewards and desirable outcomes and hence a higher tendency to engage in goal-directed behaviors (Gray, 1991). The BAS scale included in this study is the sum of three subscales: BAS drive, BAS fun seeking, and BAS reward responsiveness. Taken together, these measures represent a sufficient set of variables that reflect interindividual differences in inhibitory control and reward sensitivity.

### MRI Data Acquisition

The resting-state fMRI acquisition parameters are described in full detail in Mendes et al. (2019). In summary, four resting-state fMRI scans were acquired for each individual in axial orientation using T2*-weighted gradient-echo echo planar imaging (GE-EPI) with multiband acceleration. Sequences were identical across the four runs, with the exception of varying slice orientation and phase-encoding direction. The phase-encoding direction was anterior–posterior (AP) for Runs 1 and 3, and posterior–anterior (PA) for Runs 2 and 4. The complete set of parameters was set as follows: voxel size = 2.3-mm isotropic, FOV = 202 × 202 mm^2^, imaging matrix = 88 × 88, 64 slices with 2.3-mm thickness, TR = 1,400 ms, TE = 39.4 ms, flip angle = 69, echo spacing = 0.67 ms, bandwidth = 1,776 Hz/Px, partial Fourier 7/8, no pre scan normalization, multiband acceleration factor = 4,657 volumes, duration = 15 min 30 s per run. Individuals were instructed to remain awake, during the resting-state scan, with their eyes open and to fixate on a crosshair. The use of four 15-min rs-fMRI scans per subject enhances the temporal signal-to-noise ratio (tSNR) in the data, improves the estimation of resting-state networks, and permits a more reliable modeling of functional connectivity dynamics in the brain.

### Resting-State fMRI Preprocessing

The preprocessing pipeline is described in full detail in Mendes et al. (2019). In summary, the preprocessing steps included (a) removal of the first five volumes from each of the four resting-state fMRI runs, (b) rigid-body alignment to the first volume using FSL MCFLIRT to obtain transformation parameters for motion correction; (c) fieldmap unwarping using FSL-FLIRT and FSL-FUGUE to estimate transformation parameters for distortion correction (Jenkinson, Beckmann, Behrens, Woolrich, & Smith, 2012); (d) coregistration to each subject’s structural scan via FreeSurfer’s boundary-based registration to estimate transformation parameters for coregistration; (e) normalization of structural scans to MNI152 2-mm space using diffeomorphic nonlinear registration as implemented in ANTsSyN algorithm to estimate transformation parameters for spatial normalization (Avants et al., 2011) (f) application of all transformation parameters to each volume in the four resting-state runs in one interpolation step; (g) inclusion of six motion parameters, their first-order derivatives, and outliers from Nipype’s rapidart algorithm as nuisance regressors in a general linear model (GLM); (h) The *aCompCor* method to remove physiological noise from residual data from the previous denoising step (Behzadi, Restom, Liau, & Liu, 2007); and finally (i) band-pass filtering [0.01–0.1 Hz]. All included subjects exhibited relatively low in-scanner motion—mean framewise displacement <0.5-mm across all resting-state scans (Power, Barnes, Snyder, Schlaggar, & Petersen, 2012). Preprocessed data were obtained from https://ftp.gwdg.de/pub/misc/MPI-Leipzig_Mind-Brain-Body/derivatives/.

### Group Independent Component Analysis (GICA)

Preprocessed data from all subjects were analyzed using group independent component analysis (GICA) as implemented in the GIFT toolbox software http://mialab.mrn.org/software/gift/. GICA decomposes the rs-fMRI data into linear mixtures of spatially independent components (ICs) that exhibit unique time course profiles (Allen et al., 2014). In order to investigate cerebro-cerebellar FC, we decomposed the cerebrum and cerebellum, separately, into spatially independent components. By applying a “cerebellum-only” GICA approach, we can extract cerebellar ICs and signals that are usually overpowered by signals of cortical and subcortical origin when performing a whole-brain GICA (Dobromyslin et al., 2012). Moreover, parcellating the cerebellum permits modeling the patterns of static and dynamic FC among functionally diverse cerebellar networks and their cerebral counterparts with enhanced accuracy, relative to what is possible when considering the cerebellum as one homogeneous region of interest. Cerebral and cerebellar GICA analyses are explained in detail below.

#### Cerebellum-only GICA.

To isolate the cerebellum from the brain, we generated a cerebellar mask in MNI152 space using the standard cerebellar MNI152 anatomical template from FSL. Concatenated cerebellar rs-fMRI data across all subjects and scans were demeaned and analyzed using principal components analysis (PCA) to reduce the dimensionality to 100 subject-level PCs (retaining > 99% of the variance in the data) and 25 group-level PCs. Then, we applied the Infomax algorithm 20 times using the ICASSO toolbox to automatically estimate and select the most reliable set of 25 independent components in the cerebellum. We then used the group information guided ICA, or GIG-ICA, to estimate subject-specific ICs and time series. GIG-ICA extracts subject-specific ICs and time series with better accuracy and correspondence than dual regression (Salman et al., 2019). The choice of number of components was in accordance with previous studies that identified between 7 and 20 cerebellar RSNs using different data-driven techniques (Bernard et al., 2012; Buckner et al., 2011; Kipping, Tuan, Fortier, & Qiu, 2016; Wang, Kipping, Bao, Ji, & Qiu, 2016). However, since noise may still be present in the data even after preprocessing, we assumed a slightly higher number of ICs than the putative number of cerebellar RSNs to allow for better disentanglement of signals from each other and from noise. ICs that exhibited spatial activation near the gray matter/white matter/cerebro-spinal fluid borders or exhibited irregular patterns with no functional relevance were discarded as noise, whereas ICs that exhibited unilateral/bilateral spatial activation in the gray matter or had relevance to well-known cerebellar functional clusters were retained as RSNs (Buckner et al., 2011). Finally, the time series of cerebellar RSNs were standardized to have a mean equal to 0 and a standard deviation equal to 1 for each subject in each resting-state run.

#### Cerebral GICA.

A similar approach to the cerebellum-only GICA was performed in order to estimate cerebral RSNs. A brain mask in MNI152 space was generated from a standard FSL MNI152 brain anatomical template after setting all cerebellar voxels to 0 (Jenkinson et al., 2012). Concatenated, demeaned data from all subjects and runs were analyzed using PCA to estimate 120 subject-level PCs (retaining > 99% of the variance in the data) and, subsequently, 30 group-level PCs. We applied the Infomax algorithm 20 times using ICASSO to estimate and automatically select the most reliable set of 30 ICs, and GIG-ICA to estimate subject-specific ICs and time series. The choice of the number of components was driven by our interest in large-scale brain networks that were suitable for subsequent FC analysis in terms of dimensionality, complexity, and interpretability. ICs that exhibited spatial activation near the edges and in the white matter were discarded. Finally, the time series of cerebral RSNs were standardized to have a mean equal to 0 and a standard deviation equal to 1 for each subject in each resting-state run.

### Functional Connectivity Analysis

#### Static functional connectivity.

To construct static FC matrices, we computed pairwise Pearson’s full and partial correlation coefficients in each of the four resting-state fMRI runs using the Ledoit-Wolf estimator as implemented in the nilearn and scikit-learn Python packages (Abraham et al., 2014; Ledoit & Wolf, 2004). Pearson’s full and partial correlation coefficients quantify and reflect different types of FC: Full correlations measure direct and indirect functional connections, whereas partial correlations measure direct functional connections only (Varoquaux & Craddock, 2013). This is informative because cerebro-cerebellar networks form closed-loop circuits, and it is likely that cerebellar networks directly connect to singular brain networks rather than affecting large-scale complex processes. However, indirect connections are also believed to expand the influence of the cerebellum, according to Sokolov, Miall, and Ivry (2017).

The constructed FC matrices were Fisher r-to-z transformed to stabilize the variance of correlation coefficients and corrected for the effective number of degrees of freedom according to Bartlett’s theory, which controls for the effect of serial autocorrelation on the estimation of FC (Afyouni, Smith, & Nichols, 2019; Bartlett, 1946). Then, for each resting-state run, we extracted the cerebro-cerebellar FC subnetwork and calculated the weighted degree or strength of cerebral RSNs of interest as the sum of their positively weighted cerebellar edges, and averaged the values across all resting-state runs for each subject. This metric reflected the total static influence of the cerebellum within distinct cerebral RSNs. The cerebro-cerebellar FC strength metric was calculated using the following formula:

$$S_{i}=\overset{J}{\underset{j=1}{\sum }}w_{ij},\;w_{ij}>0,$$ (1)

where *S*_*i*_ is the cerebro-cerebellar FC strength of cerebral RSN *i*, and *w*_*ij*_ is the weight of the edge linking cerebral RSN *i* and cerebellar RSN *j*. Negatively weighted edges were discarded because of the lack of consensus and ambiguity surrounding their nature, interpretation, and means of analysis (Hallquist & Hillary, 2018).

#### Dynamic functional connectivity.

To model whole-brain FC dynamics among the identified cerebro-cerebellar RSNs, we applied the hidden Markov models (HMMs) on temporally concatenated BOLD time series from all runs and subjects as implemented in the hidden Markov model multivariate autoregression (HMM-MAR) toolbox https://github.com/OHBA-analysis/HMM-MAR. The HMMs method is a windowless dynamic FC approach that bypasses the limitations of sliding-windows and k-means clustering by being directly applied to the BOLD time series. The method uses variational Bayesian inference to estimate a set intermittently recurring brain states at the group level, each described as a multivariate Gaussian distribution with a mean representing a spatial activation pattern and a covariance matrix representing a FC pattern (Vidaurre et al., 2017). We were mostly interested in FC changes; hence, brain states were only defined by their Pearson’s full or partial correlation matrices (i.e., FC patterns) rather than by changes in absolute signal (i.e., spatial activation). We refer to brain states as dynamic FC states in the remaining sections and subsections of the article. Partial correlation matrices were obtained by inverting the covariance matrices which were automatically regularized within the Bayesian framework (Ryali et al., 2016). Furthermore, the Bayesian inference process also permits the estimation of the probability of occurrence of each state at each time point, along with the Viterbi path that represents the most likely sequence of states (Quinn et al., 2018). These were used to estimate the subject-specific state frequency of occurrence, defined as the number of times a state is visited across all scans. The HMMs method requires a prespecified number of states, so we assumed a fixed number of 6 states as a compromise between a lower model order (5 states) and higher model orders (8, 10, and 12 states) after performing a stability analysis using all configurations (see Supplementary Figure S2). Results obtained using the 5- and 8-state configurations are presented in Supplementary Tables 2 and 3.

#### Subject-specific dynamic FC matrices and temporal variability.

In order to estimate a descriptive summary measure of the dynamics of cerebro-cerebellar FC, we explored the manifestation of the states at the subject level. Particularly, we performed an additional iteration of the Bayesian inference process for each subject, given the initial group-level estimates, states probability of occurrence at each time point, and the RSNs time series as prior information when updating and reinferring the states for each subject. This yielded a maximum of 6 dynamic FC states per subject, each represented by full and partial correlation matrices. The subject-specific dynamic FC matrices were Fisher r-to-z transformed and used to calculate the state-wise strength of cerebral RSNs of interest as the sum of their positively weighted cerebellar edges, in a similar fashion to the static FC analysis. Then, we calculated the temporal variability of the strength values for each cerebral RSN *i*, denoted *V*_*i*_, as the unbiased frequency-weighted standard deviation across the states, in which the subject-specific state frequency of occurrence values were used as weight factors. This way more frequently visited states contributed more to temporal variability of FC strength. The temporal variability of FC strength was calculated using the following formula:

$$V_{i}=\sqrt{\frac{\sum_{k=1}^{6}f_{k}{\left(S_{ik}-\bar{S_{i}}\right)}^{2}}{\sum_{k=1}^{6}f_{k}-1}},$$ (2)

where *S*_*ik*_ is the cerebro-cerebellar FC strength of cerebral RSN *i* in state *k*, and the weight factor *f*_*k*_ is the frequency of occurrence of state *k*. The rationale behind computing temporal variability of cerebro-cerebellar FC using the subject-specific dynamic FC states was based on the observation that static FC matrices highly resembled the frequency-weighted mean of the dynamic FC matrices: cosine similarity > 0.98 on average for full correlation matrices and > 0.94 on average for partial correlation matrices (see Supplementary Figure S3). Therefore, static FC could be considered as a superposition of dynamic FC states identified via HMMs, as was also observed by Karapanagiotidis et al. (2018). In other words, dynamic FC states could be considered as nonrandom transient deflections from the static FC pattern at short timescales.

#### Assessing the robustness of FC dynamics.

In order to confirm the presence of robust and genuine dynamic FC in the rs-fMRI data, we generated 100 null datasets from a multivariate Gaussian distribution fitted to the rs-fMRI data of each individual subject as in Vidaurre et al. (2017). According to the authors, the correlations between brain regions in the null data are similar to those in the rs-fMRI data but are presumed to be stationary and exhibit no dynamic structure. We applied the HMMs method, with unchanged parameters, on each set of null data and extracted a metric that allowed us to compare the resultant dynamics with to those obtained in the rs-fMRI data. One important metric that has been used in a previous study is the maximum fractional occupancy (Vidaurre et al., 2017). Generally speaking, fractional occupancy (FO) is defined as the proportion of time each state is visited by each subject, whereas maximum fractional occupancy (maxFO) is the maximum proportion of time spent by each subject visiting the most occurring state (Vidaurre et al., 2017). High values of maximum fractional occupancy close to 1 indicate that a single state describes the entirety of the data and hence the absence of dynamics in FC. On the contrary, low values of maxFO indicate that multiple recurring states describe the data and hence the presence of dynamics. In this context, we compared the distributions of maximum fractional occupancy values in the rs-fMRI data with those in the null data to assess the presence/absence of genuine FC dynamics. More detailed theoretical and practical information on the use of HMMs to study brain FC dynamics can be found in Baker et al. (2014), Ryali et al. (2016), Vidaurre et al. (2017), Quinn et al. (2018), and https://github.com/OHBA-analysis/HMM-MAR/wiki/User-Guide.

### Statistical Analysis

To test for associations between cerebro-cerebellar FC and impulsivity, we used multivariate general linear models (GLMs) that included the impulsivity scales as predictors and the static and dynamic FC measures as response variables in a multiple linear regression framework. In addition, age, gender, and mean framewise displacement were included as nuisance covariates. To test for significance and correct for multiple comparisons, we applied nonparametric permutation testing with 10,000 permutations and a maximum z-statistic procedure to obtain family-wise error adjusted *p* values across all tests. Particularly, we performed random rearrangements of the labels in the observed data and estimated an empirical distribution of maximum z-statistics obtained from all tests and permutations. Then, a family-wise error adjusted *p* value for each test was obtained by computing the proportion of maximum z-scores that is above the observed *z*_0_ for each test in the nonpermuted data. This method provides strong control of Type I errors without being too conservative, as is the case with conventional techniques that correct for multiple comparisons (e.g., Bonferroni; Winkler, Ridgway, Webster, Smith, & Nichols, 2014). We reported significant associations with family-wise error adjusted *p* < 0.05.

Furthermore, to evaluate the replicability of our inference framework, we used repeated stratified fivefold cross-validation to split the initial sample into training (80% of data, 107 subjects) and testing (20% of data, 27 subjects) subsamples where the proportions of males and females were preserved in each split. Cross-validation was repeated 100 times with a different randomization in each repetition. This ensured that all subjects took part in the training and testing phases across all folds and repetitions. GLMs were refitted to the training data and then used to predict the outcome in the testing data. We only included the variables that exhibited significant associations in the previous step (i.e., GLMs fitted to the entire data) while controlling for age, gender, and mean framewise displacement. Finally, we reported the median values of the explained variance obtained in the training data, denoted $R_{train}^{2}$, and testing data, denoted $R_{test}^{2}$, across all folds and repetitions.

## RESULTS

### Behavioral and Demographic Data

Summary statistics of demographic and behavioral data are provided in Table 1, whereas the partial correlations between the different self-reports of impulsivity are provided in Supplementary Table 1 in the Supporting information. Variables were standardized to have a mean equal to zero and standard deviation equal to one. We assessed for possible multicollinearity using the variance inflation factor (VIF) approach. Most variables were found to have a *VIF* < 2 except for the UPPS-P negative and positive urgency subscales. Accordingly, in order to avoid potential multicollinearity effect due to the strong association between the two subscales (Student’s *t* = 11.34, *r* = 0.68, *p* < 10^−15^), we used factor analysis to obtain one urgency factor while preserving a sufficient amount of variance. The final set of self-reported impulsivity measures included six variables: UPPS-P urgency, UPPS-P lack of premeditation, UPPS-P lack of perseverance, UPPS-P sensation seeking, behavioral inhibition system (BIS) scale, and behavioral approach system (BAS) scale.

**Table 1.** Behavioral and demographic summary statistics.

	**Healthy subjects (N = 134, 62 females)**							
	Mean	*SD*	Median	Min	Max	VIF	Correlation with age (r)	Gender difference (t)
Age	24	4	25	20	40	–	–	−0.464
PosUrg	1.9	0.5	1.9	1	3	2.5	0.07	0.13
NegUrg	2.2	0.5	2.2	1.2	3.3	2.3	−0.01	2.91*
Premed	2	0.4	2	1.1	3	1.3	−0.02	−0.74
Persev	2	0.5	1.9	1	3.2	1.36	0.04	−1.54
SenSeek	2.8	0.6	2.8	1.4	4	1.13	−0.18	−3.32**
BIS	20.5	3.1	21	12	28	1.15	−0.0.1	4.7**
BAS	37.2	3.7	37	28	47	1.22	0.0.1	0.45

*Note*. ***SD***: standard deviation, **VIF**: variance inflation factor, **r**: Pearson’s correlation, **t**: student’s t, **PosUrg**: positive urgency, **NegUrg**: negative urgency, **Premed**: lack of premeditation, **Persev**: lack of perseverance, **SenSeek**: sensation seeking, **BIS**: behavioral inhibition system, **BAS**: behavioral approach system. * *p* < 0.05 , ** *p* < 0.01.

### Group Independent Component Analysis (GICA)

#### Cerebral GICA.

**Figure 1.**
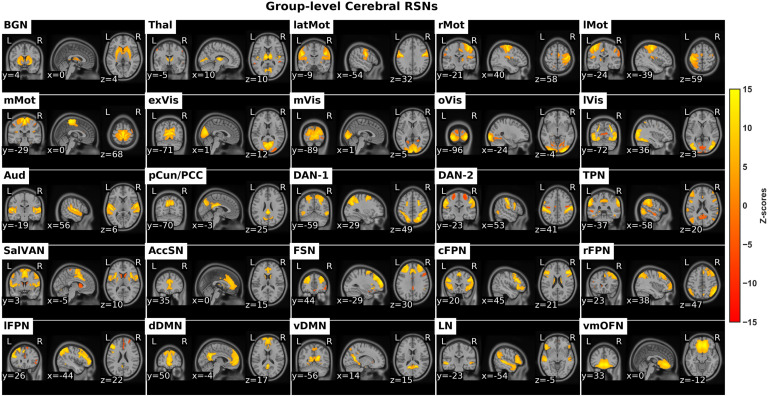
The identified cerebral RSNs. *BGN*: basal ganglia network, *Thal*: thalamus, *latMot*: lateral motor network, *rMot*: right motor network, *lMot*: left motor network, *mMot*: medial motor network, *exVis*: extra-striate visual network, *mVis*: medial visual network, *oVis*: occipital visual network, *lVis*: lateral visual network, *Aud*: auditory network, *pCun/PCC*: precuneus/posterior cingulate cortex network, *DAN*: dorsal attention network, *TPN*: task positive network, *SalVAN*: salience-ventral attention network, *AccSN*: anterior cingulate cortex salience network, *FSN*: frontal salience network, *cFPN*: central frontoparietal network, *rFPN*: right frontoparietal network, *lFPN*: left frontoparietal network, *dDMN*: dorsal default mode network, *vDMN*: ventral default mode network, *LN*: language network, *vmOFN*: ventro-medial orbito-frontal network.

Resting-state fMRI data from the cerebral cortex and subcortex were decomposed into 30 ICs out of which 25 ICs were identified as RSNs based on visual inspection of the localization of spatial activation in the gray matter (GM) and illustrated in see Figure 1. The retained RSNs were arranged into groups of putative functional groups based on their anatomical and functional properties. The functional groups were subcortical, motor, visual, auditory, attention, salience, frontoparietal, and default mode.

#### Cerebellum-only GICA.

**Figure 2.**
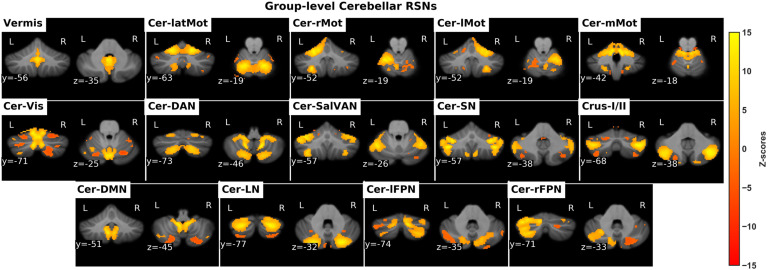
The identified cerebellar RSNs. *Cer-latMot*: cerebellar lateral motor network, *Cer-rMot*: Cerebellar right motor network, *Cer-lMot*: cerebellar left motor network, *Cer-mMot*: cerebellar medial motor network, *Cer-Vis*: cerebellar visual network, *Cer-DAN*: cerebellar dorsal attention network, *Cer-SalVan*: cerebellar salience/ventral attention network, *Cer-SN*: cerebellar salience network, *Cer-DMN*: cereballar default mode network, *Cer-LN*: cerebellar language network, *Cer-lFPN*: cerebellar left frontoparietal network, *Cer-rFPN*: cerebellar right fronto-parietal network.

Resting-state fMRI data from the cerebellum were decomposed into 25 ICs, out of which 14 ICs were visually identified as cerebellar RSNs and illustrated in Figure 2, while 11 ICs were identified as noise and hence discarded. The RSNs were arranged into groups of putative functional domains based on their anatomical or functional properties and overlap with previously established cerebellar clusters based on a winner-takes-all approach (Buckner et al., 2011). The functional clusters were motor, visual, attention, salience, frontoparietal, and default mode. However, two cerebellar RSNs, whose spatial activation maps were well situated in the GM, did not overlap with well-known cerebellar clusters. These were labelled as “Vermis” and “Crus-I/II” based on the anatomical landmarks that overlap with their spatial activation maps. In addition, taking into consideration the contralateral representation of large-scale networks in the cerebellum, labels of unilateral cerebellar RSNs were inverted. For instance, if spatial activation was mostly localized in the left posterior cerebellum, the naming would be cerebellar right frontoparietal (Cer-rFPN) because of the inverted frontoparietal map present in the posterior cerebellar lobe.

### Strength of Cerebro-Cerebellar FC and Impulsivity

**Figure 3.**
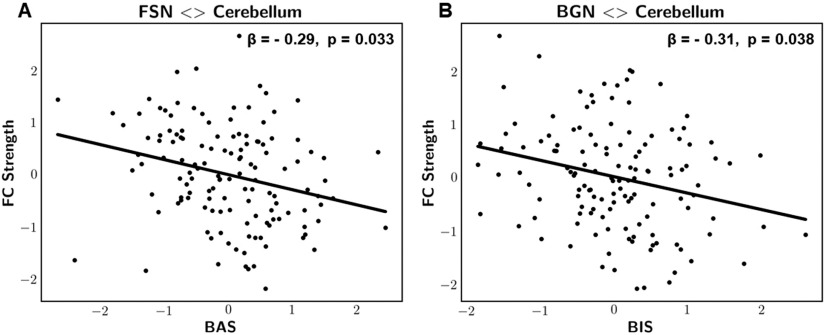
Scatterplot representations of significant associations between cerebro-cerebellar FC strength and the BIS/BAS scales. (A) FSN-cerebellum FC strength vs. behavioral approach system (BAS). (B) BGN-cerebellum FC strength vs. behavioral inhibition system (BIS). Variables were adjusted for age, gender, and mean framewise displacement and presented as z-scores. *β*: standardized regression coefficient, *p*: FWE-adjusted *p* values.

The details of statistically significant findings are reported in Table 2, whereas scatterplots are illustrated in Figure 3. Figure 5 illustrates the group-average partial correlation FC matrix and the strongest positively weighted cerebro-cerebellar connections. It clearly shows the well-documented topographic dichotomy of motor versus nonmotor cerebellum and the mostly domain-specific functional connections between cerebral and cerebellar RSNs. In accordance with our hypothesis, only static FC measures pertaining to cerebral RSNs that include regions involved in control and reward brain processes, notably the frontoparietal, salience, attention, subcortical, and default mode networks, were evaluated against self-reported measures of impulsivity using GLMs.

**Table 2.** Significant associations between cerebro-cerebellar FC and self-reported measures of impulsivity.

**Network**	**FC metric**	**Impulsivity scale**	*z*	*β*	*p*	$R_{train}^{2}$	$R_{test}^{2}$
BGN-cerebellum	Strength	BIS	−3	−0.31	0.038	0.07	0.068
	Variability	SenSeek	3.1	0.3	0.037	0.068	0.063
Thal-cerebellum	Variability	SenSeek	3.3	0.32	0.019	0.078	0.074
FSN-cerebellum	Strength	BAS	−3.1	−0.29	0.033	0.08	0.073
	Variability	Premed	−3.5	−0.34	0.008	0.092	0.09
	Variability	SenSeek	3.3	0.32	0.019	0.078	0.08
pCun/PCC-cerebellum	Variability	Premed	−3.7	−0.36	0.003	0.11	0.096
	Variability	SenSeek	3.6	0.35	0.005	0.093	0.086

*Note*. **Strength**: cerebro-cerebellar FC strength, **Variability**: temporal variability of cerebro-cerebellar FC strength, *z*: z-statistic, *β*: standardized regression coefficient, *p*: family-wise error adjusted *p* value, $R_{train}^{2}$: median of explained variance in the training data, $R_{test}^{2}$: median of explained variance in the testing data.

Statistically significant associations were observed when quantifying static FC using partial correlation coefficients. Particularly, results revealed a significant negative correlation between the behavioral inhibition system (BIS) scale and the cerebro-cerebellar strength of the basal ganglia network, BGN (*z* = −3, *β* = −0.31, *p* = 0.038, $R_{train}^{2}$ = 0.07, $R_{test}^{2}$ = 0.068). In addition, we identified a significant negative correlation between the behavioral approach system (BAS) scale and the cerebro-cerebellar strength of the frontal salience network, FSN (*z* = −3.1, *β* = −0.29, *p* = 0.033, $R_{train}^{2}$ = 0.08, $R_{test}^{2}$ = 0.073). The reported *p* values were family-wise error adjusted.

### Temporal Variability of Cerebro-Cerebellar FC and Impulsivity

**Figure 4.**
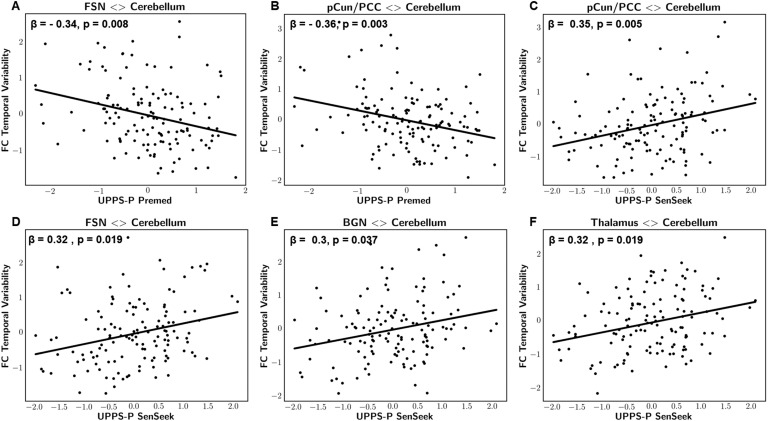
Scatterplot representations of statistically significant associations between dynamics of cerebro-cerebellar FC and UPPS-P measures: sensation seeking and lack of premeditation. Panels A and B represent temporal variability in FSN-cerebellum and pCun/PCC-cerebellum FC strength vs. UPPS-P lack of premeditation, respectively. Panels C, D, E, and F represent temporal variability in FSN-cerebellum, pCun/PCC-cerebellum, BGN-cerebellum, and thalamus-cerebellum FC strength vs. UPPS-P sensation seeking, respectively. All variables were adjusted for age, gender, and mean framewise displacement and presented as z-scores. *β*: standardized regression coefficient, *p*: FWE-adjusted *p* values.

The details of statistically significant findings are reported in Table 2, whereas scatterplots are illustrated in Figure 4. We demonstrated the inferred group-level brain FC matrices (states) along with distribution profiles of the maximum fractional occupancy values obtained from the rs-fMRI data and the generated null data in Supplementary Figure 1. The distribution profiles and the group-average frequency of occurrence values (percentages) indicated the presence of genuine FC dynamics in the rs-fMRI data as opposed to the null data, which were mostly described by a single state. In accordance with our hypothesis, only dynamic FC measures pertaining to cerebral RSNs that encompass regions involved in control and reward brain processes, notably the frontoparietal, salience, attention, subcortical, and default mode networks, were evaluated against self-reported measures of impulsivity using GLMs.

Associations were observed when describing dynamic FC states using Pearson’s full correlation matrices. Particularly, results revealed significant negative correlations between the UPPS-P lack of premeditation subscale and the temporal variability of cerebro-cerebellar FC strength of the FSN (*z* = −3.5, *β* = −0.34, *p* = 0.008, $R_{train}^{2}$ = 0.092, $R_{test}^{2}$ = 0.09) and the precuneus/posterior cingulate cortex (pCun/PCC) network (*t* = −3.7, *β* = −0.36, *p* = 0.003, $R_{train}^{2}$ = 0.11, $R_{test}^{2}$ = 0.096). In addition, results revealed significant positive correlations between the UPPS-P sensation-seeking subscale and the temporal variability of cerebro-cerebellar FC strength of the FSN (*z* = 3.3, *β* = 0.32, *p* = 0.019, $R_{train}^{2}$ = 0.078, $R_{test}^{2}$ = 0.08), the pCun/PCC network (*z* = 3.6, *β* = 0.35, *p* = 0.005, $R_{train}^{2}$ = 0.093, $R_{test}^{2}$ = 0.086), the BGN (*z* = 3.1, *β* = 0.3, *p* = 0.037, $R_{train}^{2}$ = 0.068, $R_{test}^{2}$ = 0.063), and the thalamus network (*z* = 3.3, *β* = 0.32, *p* = 0.019, $R_{train}^{2}$ = 0.078, $R_{test}^{2}$ = 0.074). The reported *p* values were family-wise error adjusted.

## DISCUSSION

In the present study, we investigated the proposed cerebellar role in impulsivity by characterizing different aspects of cerebro-cerebellar resting-state FC and testing their association with self-reported measures of impulsivity. We observed evidence that linked summary measures of static and dynamic cerebro-cerebellar resting-state FC to different forms of impulsivity in a large group of healthy subjects. Particularly, we found that the total strength and temporal variability of FC between a set of large-scale cerebral networks, involved in top-down control and reward brain processes, and the cerebellum were associated with the BIS, BAS, UPPS-P lack of premeditation, and UPPS-P sensation seeking. Nonparametric permutation testing and cross-validation based on repeated stratified fivefolds supported the significance and cross-validity of these associations, respectively. Overall, our findings improve current knowledge on the behavioral correlates of the cerebro-cerebellar system and the neural correlates of impulsivity. In addition, they highlight the utility of both static and dynamic FC analysis approaches in investigating the principles of cerebro-cerebellar coupling and the neural underpinnings of impulsivity and potentially other constructs of personality. We discuss significant findings, limitations, and future perspectives of the study in the following paragraphs.

### Cerebro-Cerebellar Static FC Strength and BIS/BAS Scales

The static FC analysis results revealed a significant negative correlation between the cerebro-cerebellar FC strength of the basal ganglia network (BGN) and the behavioral inhibition system (BIS) scale, which measures sensitivity towards unpleasant cues and punishment. This indicates that increased striato-cerebellar FC strength is associated with a lower tendency to avoid or inhibit actions with possibly negative outcomes such as punishment. We believe that this is in line with previous findings suggesting that the involvement of the cerebellum in inhibitory control mechanisms is mediated by its interaction with the basal ganglia (Brunamonti et al., 2014). In addition, we believe that this is consistent with accumulating evidence suggesting that increased connectivity between the cerebellum and the basal ganglia is associated with an overreliance on motivated “go” brain mechanisms at the expense of “no-go” inhibitory control mechanisms, and hence with increased impulsivity (Miquel et al., 2019). Results also revealed a significant negative correlation between the cerebro-cerebellar FC strength of the frontal salience network (FSN) and the behavioral approach system (BAS) scale, which measures the tendency to approach goals, rewards, nonpunishment, or escape from punishment. The FSN included parts of the dorsolateral prefrontal cortex, superior frontal cortex, fronto-polar cortex, and intraparietal cortex (or Brodmann areas BA7, BA8, BA9, and BA10) that are involved in top-down control of responses to stimuli (Corbetta & Shulman, 2002). Previous studies have proposed that the cerebellum engages with prefrontal regions in restraining ongoing actions in response to changing environmental cues, thereby promoting prefrontal functionality in top-down control of goal-directed behaviors (Miquel et al., 2019). We believe that the above-mentioned finding is in line with this proposal and indicates that stronger fronto-cerebellar FC is associated with lower activation of approach behavior towards rewarding cues, and hence increased top-down control of goal-directed behaviors.

**Figure 5.**
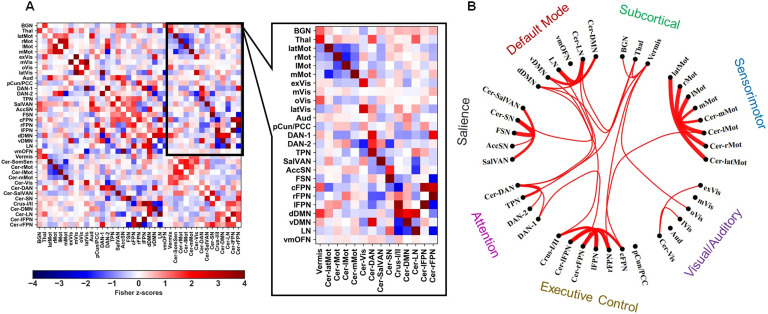
(A) The group-average partial correlation static FC matrix and the cerebro-cerebellar submatrix. (B) A circular graph showing the strongest 20% of direct cerebro-cerebellar connections in the group-average static FC matrix.

The associations between cerebro-cerebellar FC strength and the BIS/BAS scales were observed when quantifying static FC using partial correlation coefficients. Partial correlation matrices are sparse representations of FC in the brain, and they may relate to the underlying structural connectivity better than full correlation coefficients (Varoquaux & Craddock, 2013). In this case, the cerebro-cerebellar FC strength of a cerebral RSN is the sum of its positively weighted direct links to the cerebellum, and hence might be related to the parallel closed-loop cerebro-cerebellar circuitry. This is illustrated in Figure 5, which clearly shows significantly stronger within–functional domain cerebro-cerebellar connections than between domains at the group level. Even though we calculated the total strength of FC between distinct cerebral RSNs of interest and the cerebellum, the FSN was found to be mostly connected to the cerebellar salience network (Cer-SN), which overlaps with the lateral posterior cerebellum, including Crus I and II, whereas the BGN was found to be mostly connected to the cerebellar Vermis network, which mostly overlaps with the posterior lobules of the vermis. Interestingly, previous studies have shown that the lateral posterior cerebellum is mainly involved with executive control networks, whereas the posterior vermis is involved with the limbic system and connects to the ventral tegmental area (VTA), which is the center of dopaminergic cell bodies (Buckner et al., 2011; Guell et al., 2018; Miquel et al., 2019; Stoodley & Schmahmann, 2010). Moreover, lesions to the cerebellar vermis have been shown to induce an array of behavioral and emotional disturbances, including impulsiveness and behavioral disinhibition (Kim, Kim, Choi, Chung, & Moon, 2013; Miquel et al., 2019; Schmahmann, Weilburg, & Sherman, 2007). This suggests that these cerebellar structures are likely to influence processes related to control and reward by modulating prefrontal and striatal regions (Miquel et al., 2019). We believe that our findings are in line with these results, and we propose that the cerebellar influence over the fronto-striatal circuit affects motivated behaviors, in accordance with previous studies (Brunamonti et al., 2014). However, the methods used in our study are concerned with undirected FC measures that do not provide information on causal influences that neural units exert over one another. Thus, investigations using measures of directed FC, such as effective connectivity, are needed for a more comprehensive understanding of the modulatory role of different cerebellar regions in impulsivity and potentially other constructs of personality (Stephan & Friston, 2010).

### Cerebro-Cerebellar Dynamic FC and Impulsivity

Dynamic FC studies have defined temporal variability of FC as a measure of how brain regions are transiently integrated and segregated across time (Calhoun et al., 2014; Lord, Stevner, Deco, & Kringelbach, 2017). Higher values of temporal variability point to increased switching of brain FC patterns (i.e., states), which has been shown to support cognitive flexibility (Douw, Wakeman, Tanaka, Liu, & Stufflebeam, 2016). In the context of the current study, the greater the temporal variability, the more frequent the switching of FC strength between cerebral RSNs and their cerebellar counterparts. Our results revealed negative correlations between the temporal variability of cerebro-cerebellar FC strength of two top-down control networks, namely the frontal salience network (FSN) and the precuneus/posterior cingulate cortex (pCun/PCC) network, and the UPPS-P lack of premeditation subscale, which measures the tendency to act rashly without thinking. The pCun/PCC network primarily included dorsal parts of the precuneus cortex (pCun) and the dorsal posterior cingulate cortex (PCC), which is regarded as part of the executive control network and is also connected to the default mode and salience networks (Leech & Sharp, 2013; Yeo et al., 2011). This suggests that the increase in the overall switching of FC strength between the cerebellum and top-down control regions is associated with increased control of impulses. In addition, temporal variability of cerebro-cerebellar FC strength of these two top-down control networks along with two other networks involved in reward processing, namely the basal ganglia network (BGN) and the thalamus, were positively correlated with the UPPS-P sensation-seeking scale, which measures the tendency to seek new and rewarding experiences. This suggests that the increase in switching of FC strength between the cerebellum and networks involved in top-down control and reward processing is associated with enhanced salience attribution of novel experiences.

We observed associations between temporal variability of cerebro-cerebellar FC and impulsivity when using full correlation matrices to describe dynamic FC states. Pearson’s full correlation coefficients measure both direct and indirect pairwise connections that may relate to integrative brain systems involved in complex brain processes. It has been hypothesized that the cerebellum and the cerebro-cerebellar networks are key components of the integrative brain systems promoting the prediction, organization, and comprehension of complex sequences involved in higher cognitive domains (Barton, 2012; Miquel et al., 2019). In this context, our findings point to a possible dynamic system that flexibly connects multiple regions of the prefrontal cortex, precuneus, posterior cingulate cortex, basal ganglia, and thalamus with each other and with the cerebellum, and that might play a role in different elements of impulsivity. This might also be related to the postulate that the cerebellum coordinates and links cognitive units of thought that arise from multiple brain regions in a similar fashion to coordinating multimuscled movements, hence facilitating cognitive processing (Buckner, 2013). In other words, cerebellar modules might be dynamically recruited to help ensure smooth and coordinated information flow within and between top-down control and reward networks, hence influencing complex processes that underlie different forms of impulsivity. However, since we analyzed the dynamics of undirected FC and only reported correlations with impulsivity, the interpretations of the current findings remain speculative. Thus, a causal link between the dynamics of cerebro-cerebellar FC and impulsivity cannot be concluded in this case, and extensive investigations are definitely needed in the future. However, given the body of evidence linking dynamic FC to cognitive flexibility and learning, and the universal principle of cerebellar functioning in adaptive control and prediction, we believe that investigating the dynamics of cerebro-cerebellar functional networks has the potential of enhancing our understanding of the principles of cerebellar functioning in the cognitive domains (Bassett et al., 2011; Douw et al., 2016; Sokolov et al., 2017).

### Limitations and Future Perspectives

There are several limitations worth noting in the current study. A first limitation, we believe, is the use of low-dimensional GICA decomposition of the cerebrum and cerebellum that only estimated large-scale RSNs. Although this choice served our goals and interests, it was driven by the fact that computing partial correlation coefficients from high-dimensional data is computationally challenging, and the fact that the HMMs method may not converge reliably when the number of considered brain regions is high (Karapanagiotidis et al., 2018; Varoquaux & Craddock, 2013). High-dimensional representations of the brain decompose large-scale RSNs further into subnetworks and possibly aid in exploring associations between left versus right cerebro-cerebellar networks and impulsivity. Developing new methods that can reliably process high-dimensional data is needed in order to overcome this limitation. A second limitation is the sole use of positively weighted edges to estimate the strength and temporal variability of FC while discarding negatively weighted edges. Negatively weighted edges are often not analyzed because of the ambiguity and controversy surrounding their nature and means of analysis (Hallquist & Hillary, 2018). In addition, the importance of negative edges in cerebro-cerebellar FC is still unknown. Thorough investigations into the nature of negative FC and its relation to cerebro-cerebellar coupling should be conducted in future research. A third limitation of this study is the lack of objective measurements of impulsivity such as tasks (e.g., go/no-go task, stop-signal task, reward devaluation tests), which could have provided another perspective with less bias than self-reported measures. Finally, future investigations into the role of the cerebellum in impulsivity and other related traits should explore gender and age differences and include individuals diagnosed with neuropsychiatric disorders, such as alcohol-use disorder and attention deficit hyperactivity disorder, that are known to feature impulsive and compulsive symptomatology and exhibit alterations in the cerebro-cerebellar system.

## ACKNOWLEDGMENTS

The authors thank Joel Swendsen, Diego Vidaurre, Alexander Schäfer, Soroush Afyouni, and Judy Kipping for valuable advice and recommendations.

## SUPPORTING INFORMATION

Supporting information for this article is available at https://doi.org/10.1162/netn_a_00149.

## AUTHOR CONTRIBUTIONS

Majd Abdallah: Conceptualization; Formal analysis; Investigation; Methodology; Software; Visualization; Writing - Original Draft; Writing - Review & Editing. Nicolas Farrugia: Data curation; Formal analysis; Methodology; Supervision; Validation; Writing - Review & Editing. Valentine Chirokoff: Data curation; Investigation. Sandra Chanraud: Funding acquisition; Project administration; Supervision; Validation; Writing - Review & Editing.

## FUNDING INFORMATION

Sandra Chanraud, Translational Research and Advanced Imaging Laboratory (TRAIL), Award ID: TRAIL-ANR-10-LABX-57.

## TECHNICAL TERMS

Inhibitory control: The ability to inhibit unwanted habitual behaviors in order to perform a task or complete a certain goal.
Reward sensitivity: The tendency to detect, seek, and derive pleasure from rewarding and desirable stimuli.
Behavioral disinhibition: The lack of restraint or control over one’s actions or behaviors commonly triggered by brain injury.
Cerebro-cerebellar system: The set of highly functionally and anatomically interconnected cerebral and cerebellar regions.
Domain-general function: The uniform function of adaptive control or error-based learning attributed to the cerebellum across motor and cognitive domains.
Large-scale brain network: A collection of widespread brain regions having strong functional connectivity and performing the same function.
Hidden Markov models: A probabilistic method to analyze sequential data (e.g., time series) and find hidden patterns (i.e., states) that underlie them.
Direct functional connections: Functional connectivity between any two brain regions after statistically removing the effect of all other regions.
Closed-loops circuits: The set of neuronal connections that carry signals from cerebral regions to the cerebellum and back to the same regions.
Top-down control: The process of guiding and directing behavior towards a goal or in response to a changing environment.
