# Supplementary material for: Static and dynamic aspects of cerebro-cerebellar functional connectivity are associated with self-reported measures of impulsivity: A resting-state fMRI study
Source: Netw Neurosci. 2020 Sep 1;4(3):891–909. doi: 10.1162/netn_a_00149 (PMC7888486; doi:10.1162/netn_a_00149)
Supplement: Supplementary file 1 [file netn-04-891-s001.pdf]

Abdallah, M., Farrugia, N., Chirokoff, V., Chanraud, S. (2020). Supporting information for "Static and Dynamic Aspects of Cerebro-Cerebellar Functional Connectivity are Associated with Self-reported Measures of Impulsivity: A Resting-State fMRI Study. " *Network Neuroscience, 4*(3), 891-909. https://doi.org/10.1162/netn_a_0049

Supplementary Material

1 Relationship Between Impulsivity Scores

Table 1: Correlations Among Impulsivity Scales Assessed by Multiple Linear Regression

	PosUrg	NegUrg	PreMed	Persev	SenSeek	BIS	BAS
PosUrg	1	0.63***	0.16	0.16	0.00	-0.03	0.10
NegUrg		1	0.05	0.08	-0.03	0.3**	-0.02
PreMed			1	0.28***	0.23**	-0.23**	0.00
Persev				1	0.04	0.16	-0.37***
SenSeek					1	-0.13	0.25**
BIS						1	-0.00
BAS							1

Abbreviations *PosUrg*: Positive Urgency, *NegUrg*: Negative Urgency, *Premed*: Lack of Premeditation, *Persev*: Lack of Perseverance, *SenSeek*: Sensation Seeking, *BIS*:Behavioral Inhibition System, *BAS*: Behavioral Approach System.

$*p < .05$, $**p < .01$,$***p < .001$

2 Dynamic FC Analysis

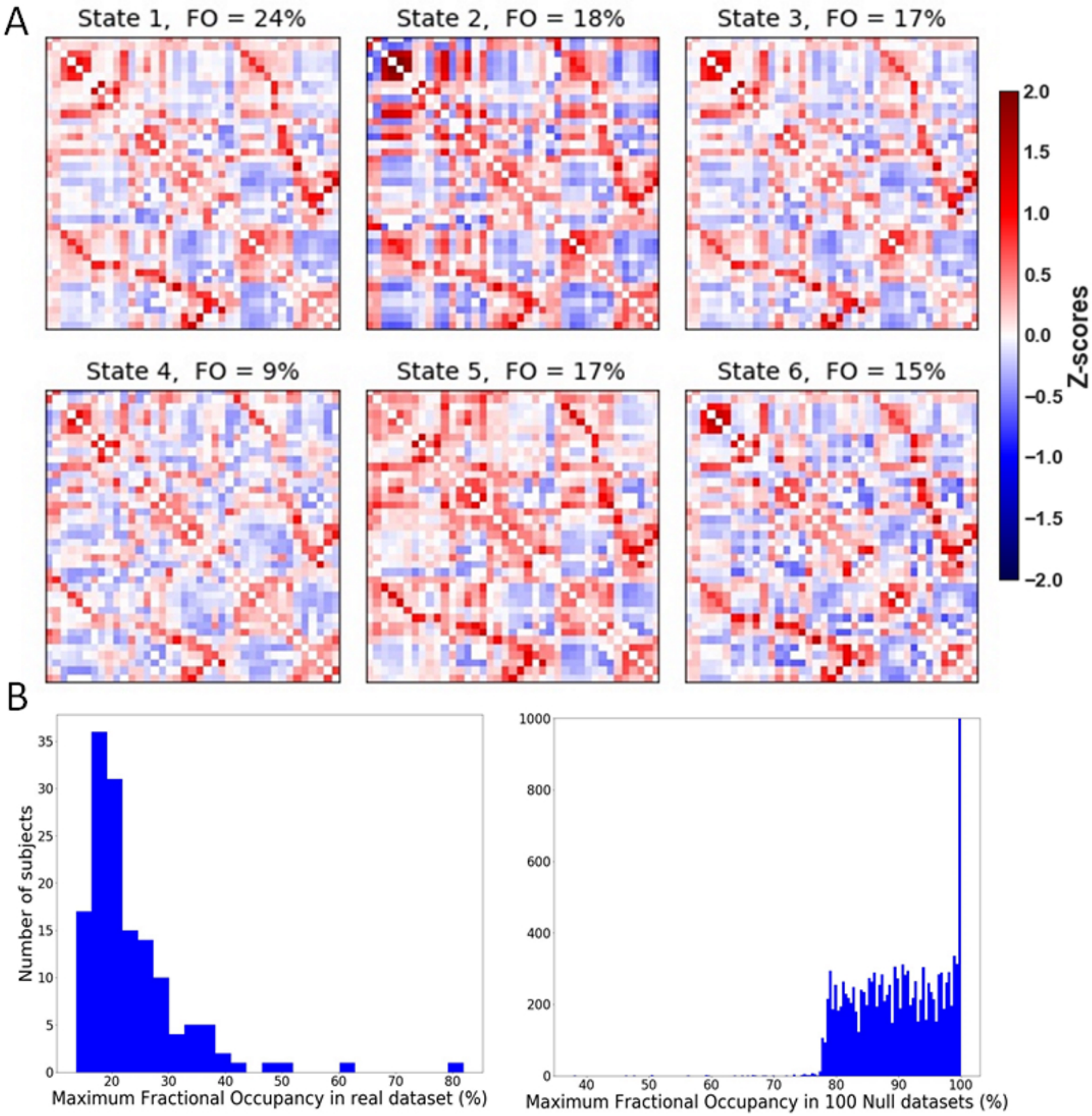

Figure 1: Dynamic FC analysis results. A) Group level dynamic FC patterns (states). At the top of each matrix is the group average fractional occupancy (FO) which represents the percentage of time each state is visited on average. B) Maximum fractional occupancy distributions in the real rs-fMRI dataset and the null datasets. The profiles of both distributions indicate the presence of genuine dynamics in the real rs-fMRI data.

3 Hidden Markov model stability analysis

Previous studies that have used hidden Markov modelling of brain FC dynamics roughly estimated between 5 and 12 brain states (Baker et al., 2014; Ou et al., 2015; Vidaurre et al., 2017, 2018; Kottaram et al., 2019; Karapanagiotidis et al., 2018). In this study, we considered testing the stability of models with 5, 6, 8, 10, and 12 states by repeating the Bayesian inference 100 times in each case and then estimating the degree of similarity between the states time courses across multiple runs, measured as the amount of overlap between state probabilities when the states are aligned via the Hungarian algorithm (Munkres, 1957). This was performed because the inference process can converge to different solutions in each run, and higher similarity between states time courses across runs indicates a higher level of stability (Karapanagiotidis et al., 2018). As expected, models with lower number of states were more stable across runs than models with a higher number of states. However, models with a higher number of states attained lower levels of the free-energy index. Accordingly, we decided, as a compromise between stability, free-energy index, and temporal resolution, to use the 6 states model which was significantly more stable than the 8, 10 and 12 states models and attained a lower free-energy index than the 5 states model. Out of the 100 runs using the 6 states model, we chose the optimal solution that corresponded to the lowest free energy index as the final estimate of group-level dynamic brain FC states. Figure 2 illustrates the similarity (or stability) matrices that encode levels of pair-wise overlap between 100 runs of the HMM using 5 different configurations. High similarity values closer to 1 indicate more stable and reliable results across multiple runs of the algorithm. Runs were re-ordered in ascending order of attained free-energy index. All code used to execute this analysis can be found in `https://github.com/OHBA-analysis/HMM-MAR`.

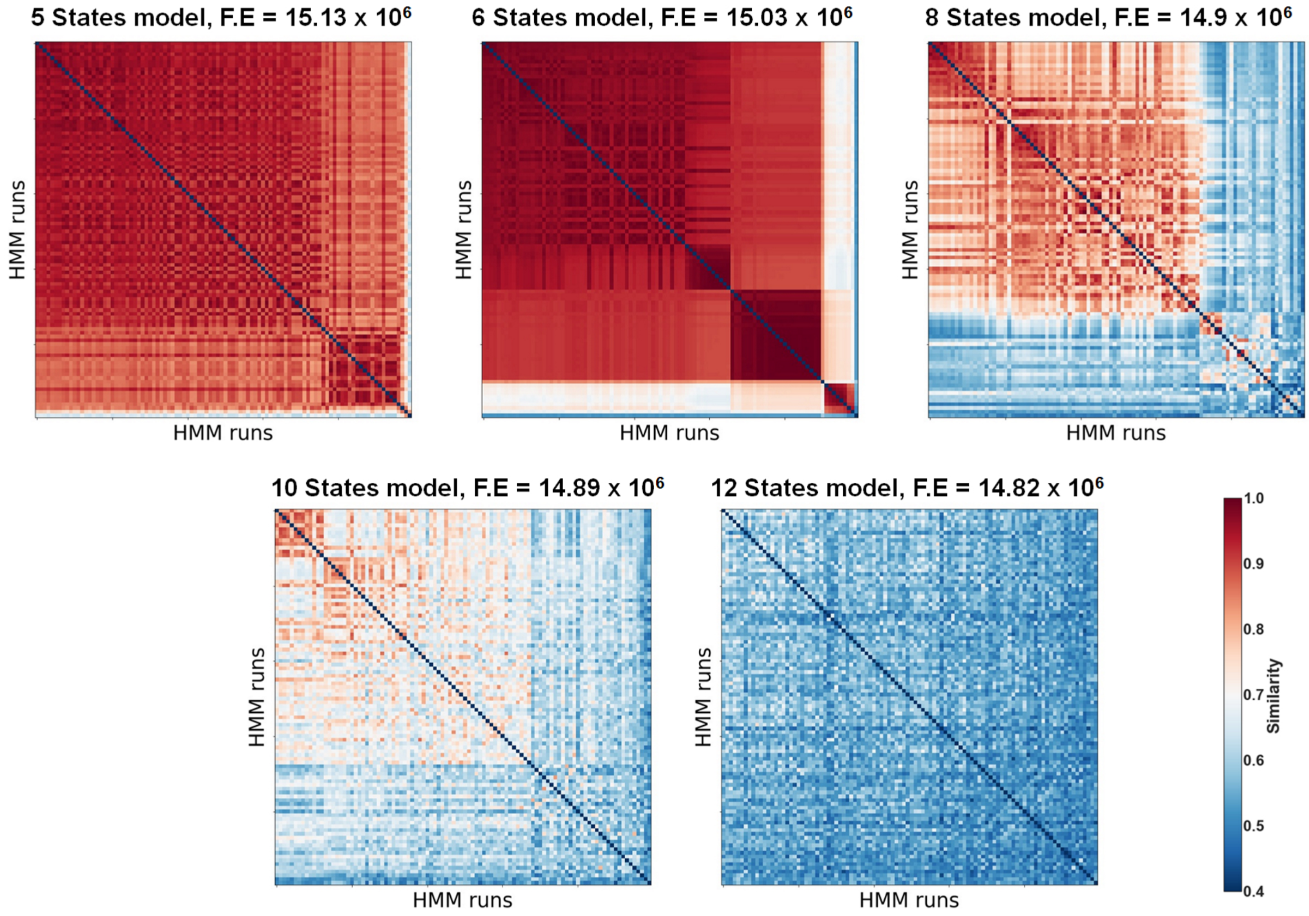

Figure 2: Similarity between 100 runs of HMM in 5 different configurations. F.E. stands for average free energy index across runs.

4 Similarity between Static and Dynamic FC matrices

The rationale behind computing temporal variability of cerebro-cerebellar FC using the subject-specific dynamic FC states was based on the observation that static FC matrices highly resembled the frequency-weighted mean of the dynamic FC matrices: cosine similarity > 0.98 on average for full correlation matrices and > 0.94 on average for partial correlation matrices. A bar chart showing the cosine similarity level between the static FC matrix (full correlation) and the frequency-weighted mean of the dynamic FC matrices (full correlation) at the subject-level is illustrated in figure 3 below.

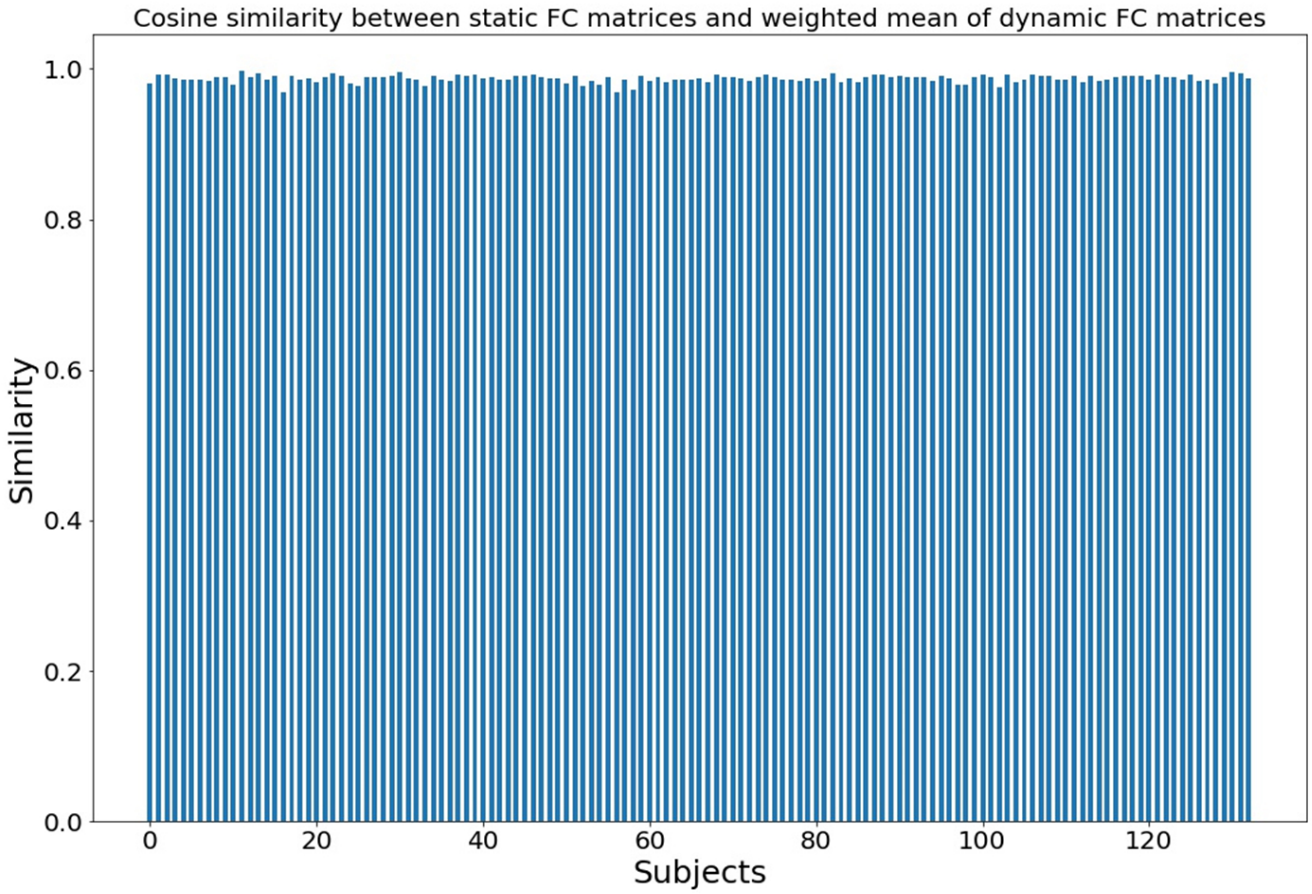

Figure 3: Cosine similarity between the static FC matrix and the frequency-weighted mean of the dynamic FC matrices for each subject (Mean= 0.986)

5 Results obtained using the 5 and 8 states HMM configurations

The results obtained using the 5 and 8 states HMM configurations reflected similar patterns of associations as those observed when using the 6 states configuration. Particularly, significant results were observed only when using full correlation matrices to describe the dynamic FC states. The statistical details of significant associations are reported in the Tables **??** and 2 below. Note that we did not correct for multiple comparisons in this validation step.

Table 2: Results obtained using the 5 states HMMs configuration

Cerebral RSN	Impulsivity Scale	z	β	p-value
FSN	Premed	-3.4	-0.33	0.001
	SenSeek	3.1	0.31	0.002
pCun/PCC	Premed	-3.6	-0.34	$<.001$
	SenSeek	3.6	0.36	$<.001$
BGN	SenSeek	3	0.29	0.003
Thal	SenSeek	2.8	0.28	0.005

Table 3: Results obtained using the 8 states HMMs configuration

Cerebral RSN	Impulsivity Scale	z	β	p-value
FSN	Premed	-2.1	-0.22	0.045
	SenSeek	2.5	0.26	0.027
pCun/PCC	Premed	-2.9	-0.28	0.007
	SenSeek	3.6	0.36	$<.001$
BGN	SenSeek	2.6	0.27	0.008
Thal	SenSeek	3.1	0.31	0.002

References

Baker, A. P., Brookes, M. J., Rezek, I. A., Smith, S. M., Behrens, T., Smith, P. J. P., and Woolrich, M. (2014). Fast transient networks in spontaneous human brain activity. *Elife*, 3:e01867.

Karapanagiotidis, T., Vidaurre, D., Quinn, A. J., Vatansever, D., Poerio, G. L., Jefferies, E., Margulies, D. S., Nichols, T. E., Woolrich, M. W., and Smallwood, J. (2018). Neural dynamics at rest associated with patterns of ongoing thought. *bioRxiv*, page 454371.

Kottaram, A., Johnston, L. A., Cocchi, L., Ganella, E. P., Everall, I., Pantelis, C., Kotagiri, R., and Zalesky, A. (2019). Brain network dynamics in schizophrenia: Reduced dynamism of the default mode network. *Human brain mapping*, 40(7):2212–2228.

Munkres, J. (1957). Algorithms for the assignment and transportation problems. *Journal of the society for industrial and applied mathematics*, 5(1):32–38.

Ou, J., Xie, L., Jin, C., Li, X., Zhu, D., Jiang, R., Chen, Y., Zhang, J., Li, L., and Liu, T. (2015). Characterizing and differentiating brain state dynamics via hidden markov models. *Brain topography*, 28(5):666–679.

Vidaurre, D., Abeysuriya, R., Becker, R., Quinn, A. J., Alfaro-Almagro, F., Smith, S. M., and Woolrich, M. W. (2018). Discovering dynamic brain networks from big data in rest and task. *NeuroImage*, 180:646 – 656. Brain Connectivity Dynamics.

Vidaurre, D., Smith, S. M., and Woolrich, M. W. (2017). Brain network dynamics are hierarchically organized in time. *Proceedings of the National Academy of Sciences*, 114(48):12827–12832.
